# Sex-specific transgenerational effects on murine thyroid gland imposed by ancestral exposure to neonicotinoid thiacloprid

**DOI:** 10.1038/s41598-024-63986-w

**Published:** 2024-06-06

**Authors:** Mariam Diba Lahmidi, Morgane Le Noc, Ouzna Dali, Pierre-Yves Kernanec, Pierre-Etienne Merret, Christian Jaulin, Fatima Smagulova

**Affiliations:** 1 Université de Rennes, EHESP, Inserm, Irset (Institut de Recherche en Santé, Environnement et Travail) - UMR_S 1085, 35000 Rennes, France; 2 Irset-Inserm UMR 1085, 9 Avenue du Prof. Léon Bernard, 35000 Rennes, France

**Keywords:** Thiacloprid, Transgenerational inheritance, Thyroid gland, Histone H3K4me3, Epigenetic memory, Developmental biology

## Abstract

Neonicotinoids, a relatively new widely used class of insecticide is used in agriculture to control insect populations. We examined the capacity of ancestral exposure to the neonicotinoid *thiacloprid (thia)* to induce transgenerational effects on thyroid tissue. Pregnant outbred Swiss female mice were exposed to *thia* at embryonic days E6.5 to E15.5 using 0, 0.6, and 6 mg/kg/day doses. Thyroid paraffin sections were prepared for morphology analysis. We apply ELISA method to measure T4 and TSH levels, RT-qPCR for gene expression analysis, ChIP-qPCR techniques for sperm histone H3K4me3 analysis, and immunofluorescence microscopy and western blots for protein detection. We observed an alteration in the morphology of thyroids in both males and females in the F3 generation. We observed an increase in T4 hormone in F1 females and a significant T4 level decrease in F3 males. T4 changes in F1 females were associated with a TSH increase. We found that the amount of Iodothyronine Deiodinase 1 (DIO1) (an enzyme converting T4 to T3) was decreased in both F1 and F3 generations in female thyroids. GNAS protein which is important for thyroid function has increased in female thyroids. Gene expression analysis showed that the expression of genes encoding thyroid gland development, chromatin, biosynthesis and transport factors were affected in the thyroid gland in both sexes in F1 and F3. The analysis of sperm histone H3K4me3 showed that H3K4me3 occupancy at the *Dio1* locus has decreased while Thyroglobulin (*Tg*) and Matrix Metallopeptidase 2 (*Mmp2*) genes have increased H3K4me3 occupancy in the sperm of F3 mice. Besides, DNA methylation analysis of our previously published datasets showed that, in the sperm of F1 and F3 *thia*-derived mice, several genes related to thyroid function show consistent alterations. Our data suggest that ancestral exposure to thiacloprid affects thyroid function not only in exposed but also in indirectly exposed F3 generation.

## Introduction

The thyroid gland is an important master regulator organ of all metabolic processes. Thyroid development and function involve a coordinated action of a complex network of transcription and chromatin remodelling factors and hormones. During development, thyroid cells express a specific combination of transcription factors, *Ttf1* (also known as *Nkx2-1*)^1^, *Hhex,* an early marker of the thyroid primordium^2^, *Pax8*^3,4^ and *Foxe1* (also known as *Titf2/TTF-2*)^5^. In the thyroid, *Foxe1* expression is down-regulated just before thyroglobulin (*Tg)* and thyroid peroxidase (*Tpo)* gene expression, suggesting that *Foxe1* down-regulates thyroid-specific gene expression during development^5^. It has been suggested that FOXE1 also promotes the migration of thyroid precursor cells^6^. Another factor, TBX1 is a key factor in the global development of the thyroid pharyngeal apparatus. TBX1 regulates the size of the early thyroid primordium through its expression in the adjacent mesoderm^7^. The proliferation and differentiation of thyroid epithelial cells have been found to be under the control of the thyroid stimulating hormone (TSH) that is produced in the anterior neurons of the pituitary gland^8^. Thyrocyte growth is inhibited by TGF-beta1 factor (encoded by *Tgfb1*), which increases the phosphorylation of SMAD2 and induces the nuclear translocation of SMAD2, SMAD3, and SMAD4^9^. Thus, thyroid development is coordinated by positive and negative signaling in order to restrict uncontrolled growth of the thyroid gland and proper formation of the organ.

The functional unit of the thyroid gland is the thyroid follicle, which consists of follicular cells located around a lumen containing colloid. The major function of the thyroid is the formation, storage and secretion of thyroid hormones. Thyroid hormone biosynthesis requires the iodide ion (I^−^) which is recruited in the thyroid by NIS/SLC5A5, a Na^+^/I^−^ symporter. I^−^ recruitment is the first step in thyroid hormone biogenesis at the basolateral plasma membrane of thyrocytes^10^. Thyroid hormone production requires thyroglobulin (TG) biosynthesis and its transport to the lumen of thyroid follicles. TG is a prohormone that is iodinated by thyroid peroxidase (TPO) which requires the presence of hydrogen peroxide (H2O2) that is generated by an NADPH oxidase (DUOX). TPO oxidises iodide ions in order to couple them to the TG prohormone tyrosine, leading to the production of thyroxine (T4). To release thyroid hormone, the iodinated TG is degraded via the fusion of endosomes with lysosomes. MCT8 (encoded by *Slc16a2)* transports the thyroid hormone out of the cell into the lumen^11^. T4 is converted by deiodinases 1, 2 or 3 (DIO1 and DIO2) into the T3 active form. DIO1 is expressed in thyroid, however, DIO2 is a major source of the cytoplasmic T3 pool^12^*.* Biosynthesis of thyroid hormones is regulated by thyroid-stimulating hormone (TSH) which acts via binding to its receptor (TSHR).

There are several pathologies in humans caused by perturbations in the production levels of thyroid hormones. Disruptions of thyroid morphogenesis in humans could lead to the absence of thyroid tissue, thyroid displacement, hypoplasia (small thyroid) and hemiagenesis (thyroid with a single lobe) that together are known as thyroid dysgenesis^13^. Findings from several studies suggest that defects in transcription factors functions contribute to thyroid dysgenesis^14^. It is suggested that numerous environmental factor such as bisphenols^15–17^ perfluorooctane sulfonate (PFOS)^18^ or pesticides^19^ could contribute to abnormal thyroid functions. Among those compounds are neonicotinoids which are organic molecules designed to control insect’s populations. Unlike other pesticides, they are absorbed by the plant and transported through their system. Once inside the insect, neonicotinoids act as an antagonist to the nicotinic acetylcholine receptor found in the central nervous system, overstimulating it and leading to insect paralysis^20^. It has been shown that exposure to neonicotinoid imidacloprid leads to the disruption of thyroid follicles by causing epithelial cell hypertrophy and altered colloid volume in the seasonally breeding wildlife bird, Red Munia (*Amandava amandava*)^21^. Imidacloprid exposure led to decreased plasma levels of T4 and TSH in these birds^21^. Another study performed on lizards reports that 28 days of continuous exposure to thiamethoxam induce a significant increase of plasma T3 and T4 concentrations and promotes the binding of T3 and thyroid hormone receptors^22^. Neonicotinoids also affects thyroid function in mammals: in vivo exposure during 30 days to commercial formulations of a mixture of thiacloprid and deltamethrin led to increased T3 and T4 levels in rats serum^23^. A recent epidemiological study in US adults (National Health and Nutrition Examination Survey; NHANES 2015–2016) reported the association between neonicotinoids presence in human and adiposity levels. The detectable levels of 5-hydroxy-imidacloprid were associated with greater risks of being overweight/obese^24^. The authors suggest that neonicotinoids may be associated with adiposity development via thyroid hormone disruption and increased oxidative stress^24^. Many studies supporting the role of environmental chemicals in thyroid-disrupting effects have been published and the impact of thyroid disruption in the developing organism is a serious public concern. Thus, in our study, we aimed at revealing the effects of prenatal exposure to thiacloprid on thyroid function. Moreover, we investigated whether alterations in directly exposed generations could be transmitted to next generations. In a recent study, we showed that exposure to thiacloprid (*thia)* during gestation leads to spermatogenic defects not only in the exposed embryos^25^ but also in subsequent generations^26^. Specifically, gestational exposure perturbs meiosis, causes changes in testis morphology and testosterone levels and decreases spermatozoa number in male descendants^26^.

Here we show that paternal gestational exposure to thiacloprid affects thyroid function in both sexes at the third generation. Specifically, it affects thyroid morphology, changes thyroid hormone levels, and affects gene expression. We observed that sperm histone H3K4 trimethylation were altered at several genes encoding important thyroid factors. Besides, DNA methylation patterns in the sperm of F1 and F3 were modified at the vicinity of important genes related to thyroid function.

## Results

### Experimental design

This study is aimed at revealing the transgenerational effects of *thia* exposure. We chose the developmental window from embryonic day E6.5 to E15.5 due to the importance of this period of time for germ cell lineage establishment. The design of experiments is presented in Supplementary Fig. 1. Briefly, pregnant, outbred, Swiss (RjOrl) female mice were treated with thiacloprid and control mice were treated with the same volume of oil. These control and thia-treated mice are called F0. The progeny of exposed mice is referred to as F1. Both control and exposed F1 generation males were crossed with non-littermate, untreated females to give rise to F2 generation. Similarly, both control and exposed male progenies of F2 were crossed with non-littermate and untreated females to give rise to F3 generation. We studied the animals at the age of 2 months because at this age all organs are formed, male and female mice are sexually mature, and males produce a amount of sperm suitable for the analysis.

We analysed the transgenerational effects of the three doses of *thia* “0”, “0.6” and “6” mg/kg/day on the thyroid-body weight ratios. Since in a previous study we observed that dose “0.6” had limited effects on reproductive function based on spermatozoa number, testosterone levels and meiosis defects, our analyses in this study were focused on those that were promoted by dose “6”. However, exposure to dose “0.6” led to increased thyroid weights in both sexes in F3, suggesting that this dose could also be harmful for animals and should be taken into account for a future study. We analysed thyroid morphology, thyroid hormone levels, protein levels of some thyroid factors, and performed gene expression analysis. We estimated histone H3K4me3 occupancy at several loci in spermatozoa using ChIP-qPCR, due to the importance of this mark in early embryos. Besides, we reanalysed our previously published datasets on sperm DNA methylation from F1 and F3 thia-derived mice and we report that several genes related to thyroid function show consistent alterations in both generations.

### Ancestral exposure to thiacloprid changes the thyroid size in exposed progeny

**Figure 1:**
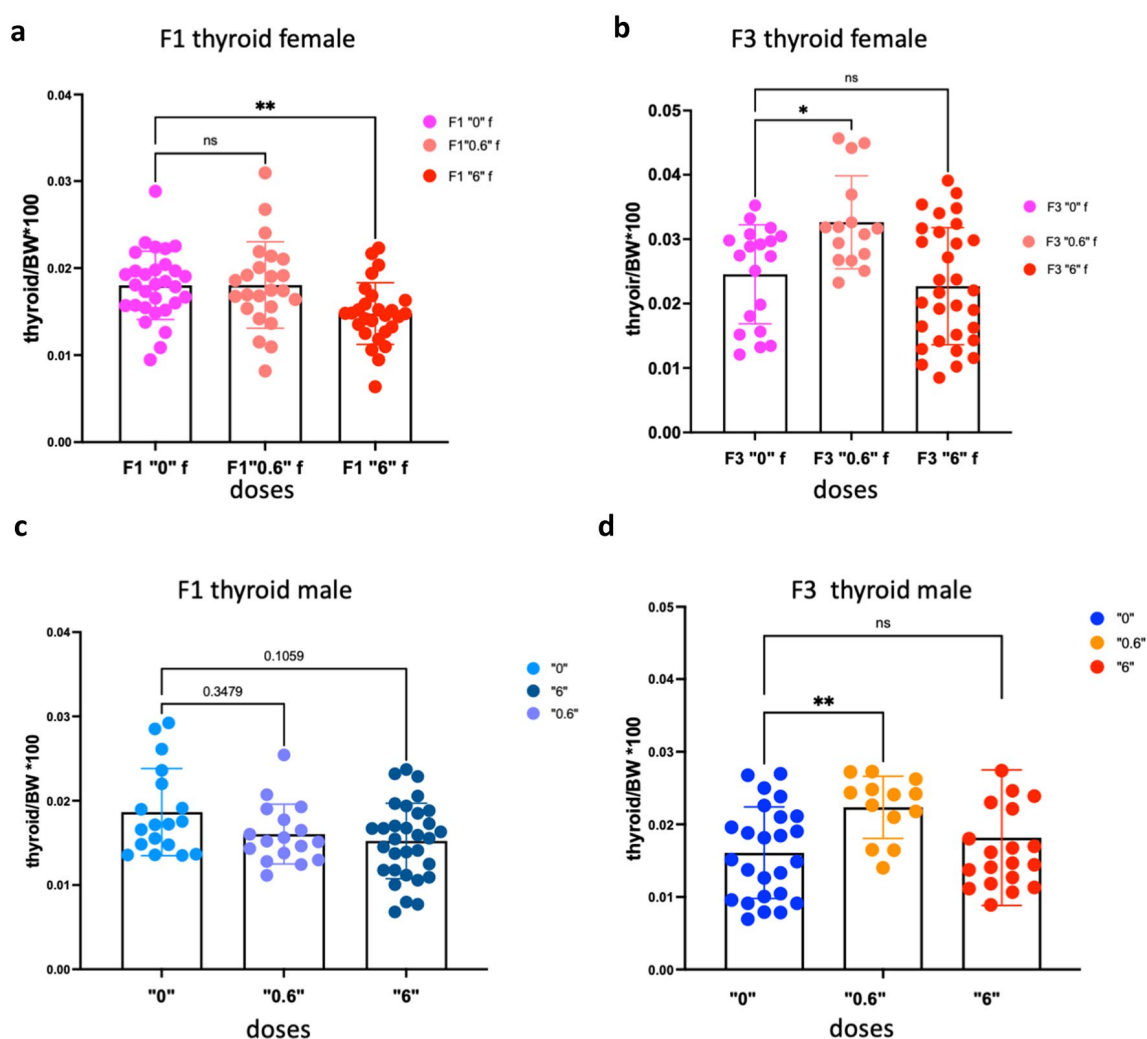
Thyroid weights were affected in females and males. Thyroids were dissected, weighted and normalised to body weight. (**a**) Normalized thyroid weights in F1 and (**b**) in F3 females, (**c**) in F1 males and (**d**) in F3 males. Non-parametric Kruskal–Wallis test was used to access the statistical significance. A minimum of 25 animals, derived from minimum 8 non-related litters, were used for each group, *p < 0.05, **p < 0.01, Mann–Whitney test.

In order to reveal the effect of *thia* exposure on thyroid morphology, we dissected thyroid gland and both lobes were weighted. Weights of both lobes were added, and the total weight of thyroid was compared to body weight. In F1 females exposed to thiacloprid we observed a significant 18% decrease in thyroid weight compared to the control (Fig. 1a). Female mice from the F3 generation, derived from dose “0.6,” show a 1.3-fold increase in thyroid weight, while female F3 mice derived from dose “6” did not show significant changes (Fig. 1b). In F1 males, the progeny derived from exposure to dose “6” show a tendency of decreases in thyroid weights (0.8-fold; *p* = 0.1) (Fig. 1c). In the F3 generation, males derived from exposure dose “0.6” show a significant increase (1.4-fold) in thyroid weight while no significant changes were detected for dose “6” (Fig. 1d).

**Figure 2:**
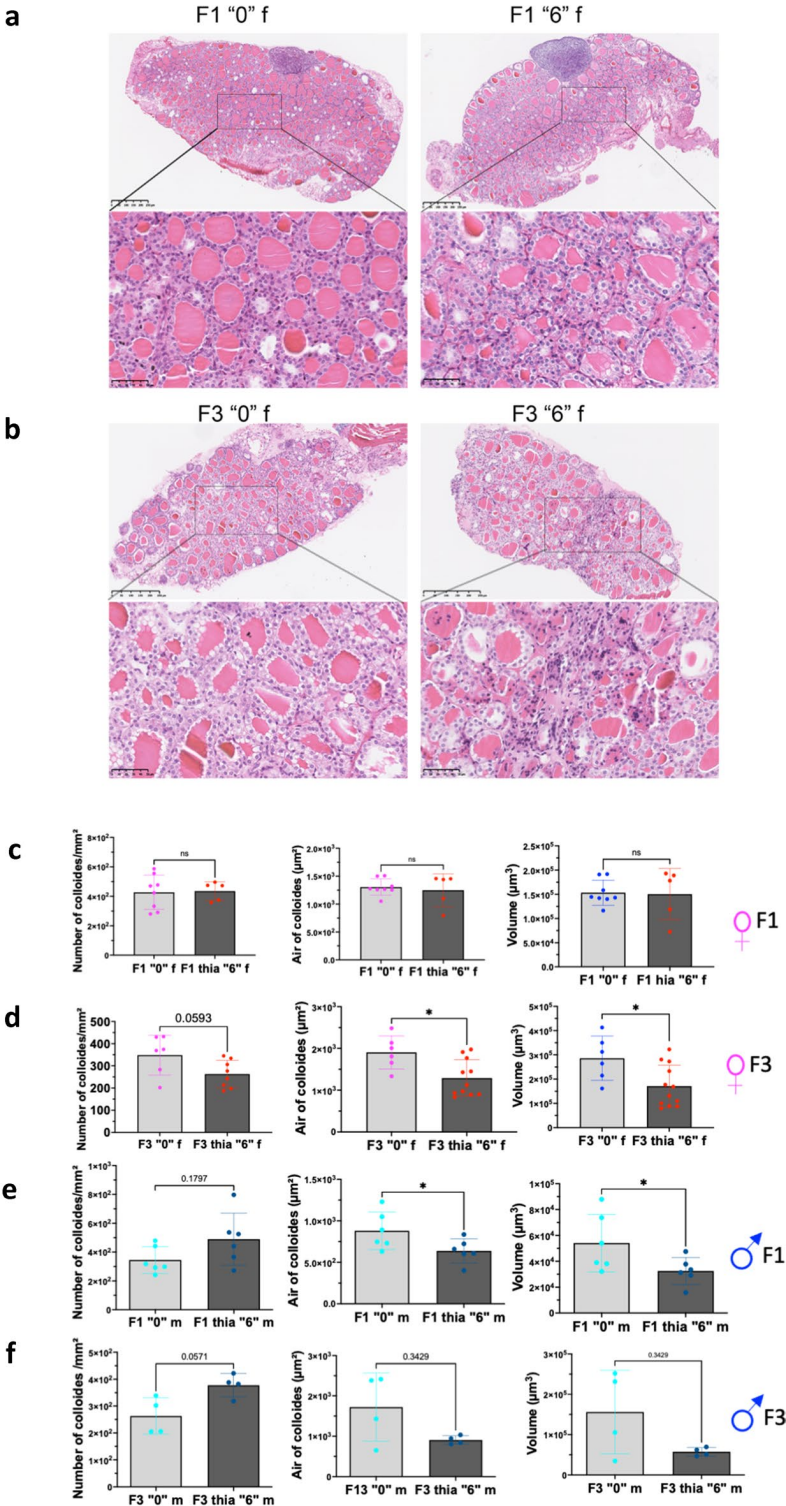
Morphological analysis of the thyroids in F1 and F3 animals. Thyroids were fixed in bouin solution and embedded in paraffin. 5 µm sections were cut and stained with hematoxylin and eosin. (**a**) A representative image of thyroid of F1 from a control (left) or *thia* “6” (right) exposed female. (**b**) A representative image of thyroid of F3 from a control (left) or *thia* “6” (right) exposed female. (**c**) Quantitative analysis of follicles in F1 and (**d**) in F3 females. (**e**) Quantitative analysis of follicles in F1 and (**f**) in F3 males. The number of follicles were counted and averaged. The volumes of follicles were measured and averaged as described in "Methods" section. A minimum of 5 animals derived from 5 different litters were used for each group, *p < 0.05, Mann–Whitney test.

To analyse morphology, the thyroids were fixed and embedded in paraffin, sections were cut and stained with Hematoxylin and Eosin (H&E). Images were taken using nanozoomer (Fig. 2a,b). The images were analysed, and the data were averaged and plotted. Specifically, we calculated the averaged size of the lumen called colloids, as well as the total volume of colloid. Our analysis showed that in F1 females no significant changes in the number of follicles, in the mean area of follicles, nor the mean volume were observed (Fig. 2c). However, we noted a large variation in the size of follicles in *thia* -derived mice compared to control mice. In F3 females, the mean area of follicles and the volume has decreased by 32% and 40% respectively in the exposed group compared to control (Fig. 2d). In F1 males, we observed a 42% increase in the number of colloids in the exposed group compared to control for dose “6”. However, this increase did not pass the significance threshold (*p* = 0.18) cut off (Fig. 2e). The area and volume of colloids in the exposed group significantly decreased by 27% and 40%, respectively, compared to the control (Fig. 2e). In F3 males, the average number of colloids increased by 43% in the exposed group compared to the control (*p* = 0.057). The mean area and volume of colloids in the exposed group decreased by 47% and 63%, respectively, compared to the control, but these changes were not significant (Fig. 2f).

To conclude, our analysis showed that ancestral exposure to thiacloprid leads to a decrease in thyroid weights in F1 females. Both males and female F3 progeny showed an increase in thyroid weights at dose “0.6”. No significant changes in thyroid weights were observed for dose “6” in F3 for both sexes. No significant changes in the morphology were observed in F1 females exposed to dose “6”. In F3 progeny, females had a significant decrease in the mean area and the mean volume of thyroid. In F1 males we observed decrease in the mean area and volume of glands and a tendency to in the number of colloids in F3.

### Thyroid hormonal imbalance in serum of F1 and F3

**Figure 3:**
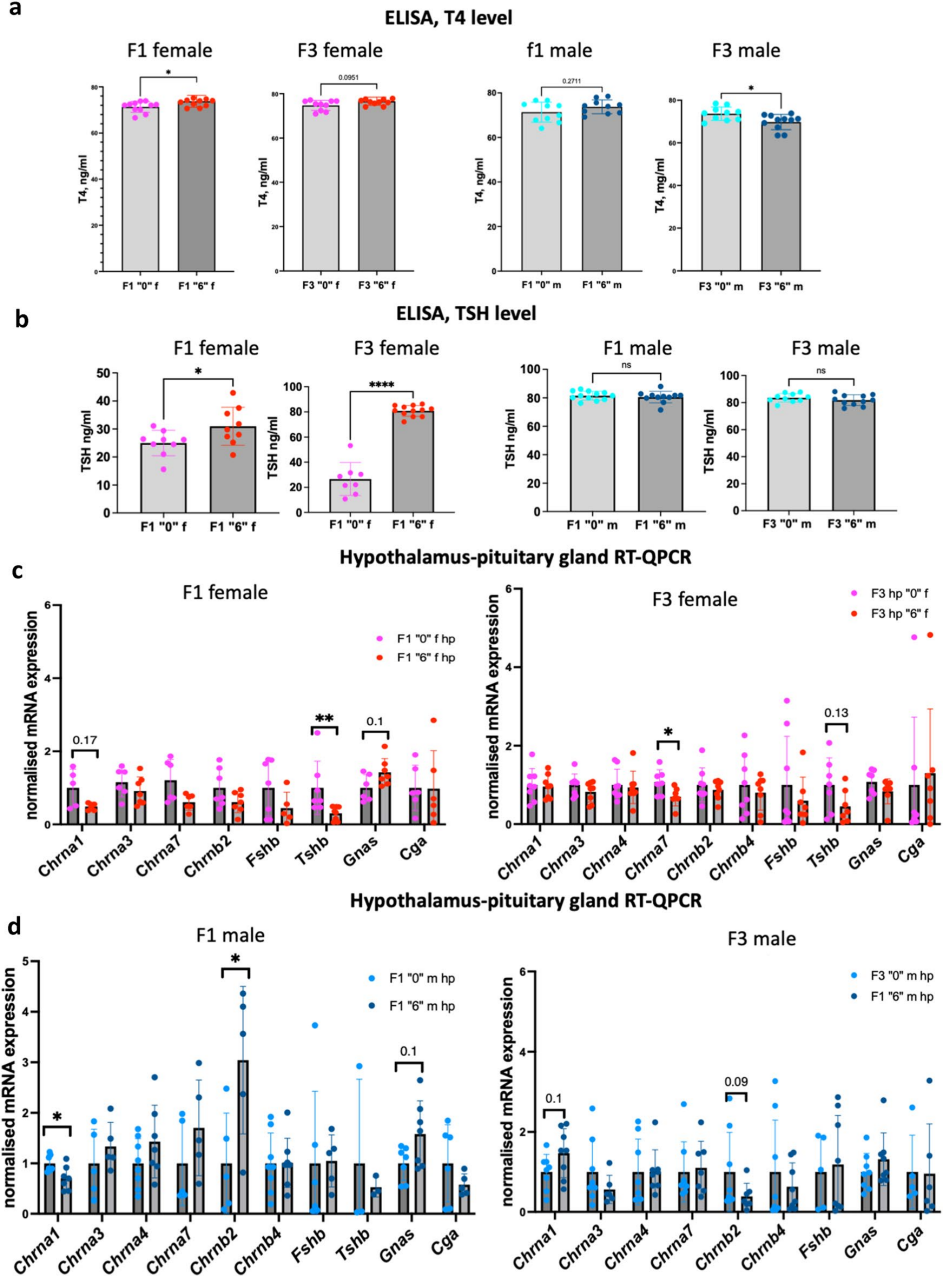
Hormone measurement in the blood serum and gene expression analysis by RT-QPCR in the hypothalamus of F1 and F3 mice. (**a**) T4 levels in F1 and F3 females (left) and in F1 and F3 males (right). Blood was collected at the moment of dissection and serum was separated from blood cells and aliquoted. (**b**) T4 and TSH levels were analyzed by ELISA. Gene expression analysis (**c**) in F1 (left) and in F3 (right) female hypothalamus. (**d**) Gene expression analysis in F1 (left) and in F3 (right) male hypothalamus. RNA was extracted and cDNA synthesized, diluted and used for qPCR. Each value was normalised to *Rpl37a*. N = 8 for each group, *p < 0.05, **p < 0.01, Mann–Whitney test.

Since the production of thyroid hormones is the major function of the thyroid, we performed the analysis of the most abundant hormone, T4, using blood serum of control and treated groups using ELISA. In F1 females, we found that T4 levels have significantly increased by 3.4% in the treated group (Fig. 3a). In the F3 generation, there is tendency to increase by 3.5% (*p* = 0.095) in the *thia* “6” derived group. In F1 males, we found an increase in T4 by 3.3% but values did not pass significant cut off (*p* = 0.27) between the two groups. In F3 males, there was a significant 5.3% decrease of T4 levels in treated mice (Fig. 3a). Because TSH is important for the regulation of T3 and T4 hormones, we decided to measure TSH by ELISA using blood serum. In F1 females, there was a significant 24% TSH increase and, in F3 females, there was a significant 304% TSH increase (Fig. 3b). No significant changes were observed in both F1 and F3 males. (Fig. 3b).

Since pituitary gland and hypothalamus plays a key role in the production of TSH and regulation of hormonal biosynthesis is also linked to the activity of the brain cholinergic system, we decided to analyse the impact of thiacloprid on gene expression in the hypothalamus and pituitary gland. To this end, we dissected hypothalamuses with the adjacent pituitary gland, extracted RNA, prepared cDNA and performed RT-qPCR analysis. We analysed genes encoding several key acetylcholine receptors such as *Chrna1, Chrna3, Chrna7,* and *Chrnb2* and *Chrnb4,* genes encoding specific subunits of TSH (*Tshb)* and FSH (*Fshb*) hormones as well as the gene encoding a common subunit of these hormones, *Cga*. Since the importance of the imprinted gene *Gnas* for hormonal regulation was reported, we also included this gene for the analysis. In F1 female brains, there was a decrease in expression of genes encoding cholinergic receptors, however the changes were not significant, in F3 females we observed a significant decrease in *Chrna7* expression (Fig. 3c). In F1 females we observed a 3.3-fold significant decrease in *Tshb*, the gene had a tendency to decrease in F3 females (Fig. 3c). In F1 males, we observed a significant decrease in *Chrna1* and significant threefold increase in *Chrnb2* expression, this gene had a tendency to decrease by 2.6-fold in F3 (Fig. 3d). Both F1 males and females had a tendency to increase in *Gnas*.

To sum up, exposure to *thia* increases T4 in F1 females suggesting that a major function of thyroid hormone production has been disrupted upon exposure in females. Both F1 and F3 females have elevated level TSH. F3 males show a decrease in T4 hormone levels, however, no significant changes in TSH were observed in both generations. The changes in thyroid hormones were associated with hypothalamus and pituitary gland gene expression alterations suggesting that hormonal disfunctions could be induced by the perturbation of nervous system regulatory mechanisms.

### Quantification of the DIO1 protein shows a decrease in F1 and F3 female thyroids

**Figure 4:**
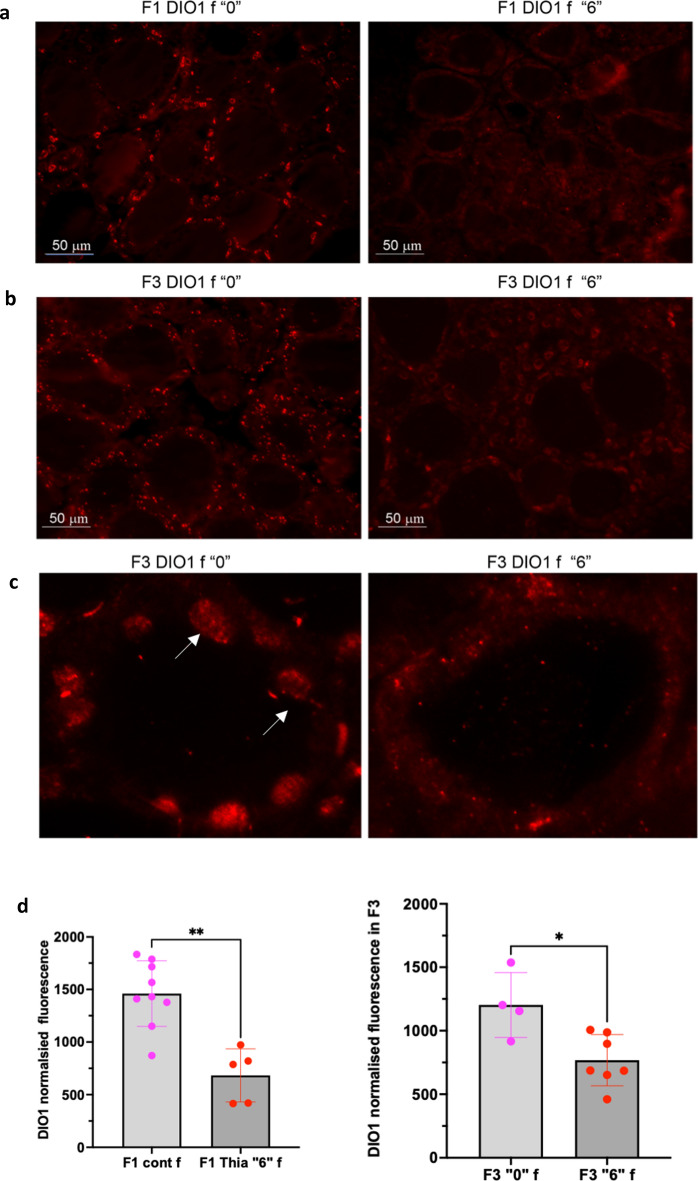
DIO1 analysis by immunofluorescence. (**a**) Representative image of the thyroid tissue immunostained by DIO1 in F1 control (left) or *thia* “6” derived female thyroid. (**b**) DIO1 in F3 female thyroids. Paraffin sections were used for incubation with DIO1 antibody followed by incubation with a secondary fluorescent antibody. The signal was quantitated for each follicle and divided by the area. (**c**) Higher magnification image (**d**). Quantitative analysis of the averaged corrected fluorescence values in F1 (left) and in F3 (right) female thyroids, n = 10 for group “0” and n = 5 for group “6” in F1 and n = 4 for group ”0″ and n = 6 for group “6″ in F3, *p < 0.05, **p < 0.01, Mann–Whitney test.

The active form of thyroid hormone is T3 which originates from the conversion of T4 by enzymes called deiodinases. Type 1 and type 2 deiodinase (DIO1 and DIO2) convert T4 into T3, whereas D3 degrades T4 and T3 into inactive metabolites. DIO1 is expressed in the thyroid, liver and kidneys and it is suggested that DIO1 is a sensitive marker of peripheral thyroid status in the mouse^27^. Thus, we decided to analyse DIO1 protein levels using immunofluorescence microscopy. We prepared thyroid tissue sections and incubated them with a primary antibody against DIO1 and a secondary fluorescent antibody. The images were taken with the same exposure time and objective aperture and the signals were measured and corrected according to the size of the analysed area. We performed this analysis only in females (Fig. 4a–c). The results showed a significant decrease of DIO1 protein in the treated group compared to the control in F1 and F3 by 53 and 37% respectively (Fig. 4d), suggesting a possible impact on T4 conversion into T3 which is consistent with the elevated T4 levels found in females.

### Changes in GNAS protein levels following *thia* exposure

**Figure 5:**
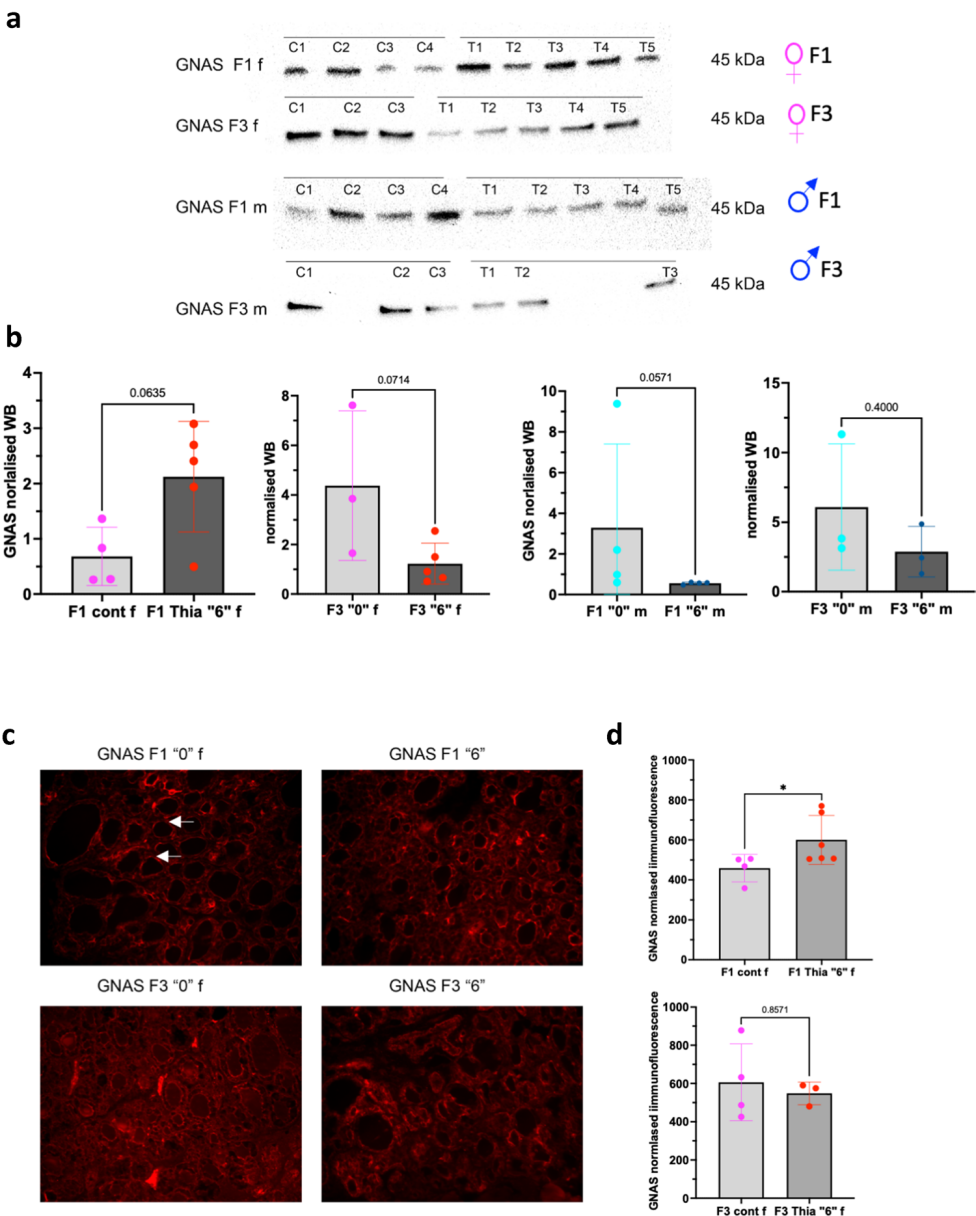
GNAS analysis by western blot and immunofluorescence in F1 and F3. (**a**) Western blot analysis. Protein extracts were prepared as described in the "Methods" section and equal amounts were loaded, transferred to a membrane and incubated with an anti-GNAS antibody. (**b**) Quantitative analysis of western blot. The signal was normalised to Ponceau Red and averaged values were plotted. The exact p-value is indicated at the top of the column. (**c**) Paraffin sections were used for incubation with anti-GNAS antibody followed by incubation with a secondary fluorescent antibody. (**d**) The signal was quantitated for each follicle and divided by the area. The averaged corrected fluorescence was plotted, n = 4 “0”, n = 6, “6” in F1 and n = 4 ”0″ and n = 3 “6″ in F3, *p < 0.05, Mann–Whitney test.

GNAS complex locus gives rise to maternally, paternally, and biallelically expressed transcripts. The gene encodes for different isoforms of the G-protein alpha subunit, which is a key element of the signal transduction pathways involving the activation of adenylyl cyclase. Since a large number of thyroid pathologies in human have been linked to GNAS deficiency^28–31^ we decided to analyse the protein levels of GNAS. First, we prepared protein extracts from thyroid tissue from several replicates and then quantified protein levels of GNAS by western blot in both female and male thyroids for both generations (Fig. 5a). Full-length gels and blots are included in a Supplementary Information file (Supplementary Fig. S2). The analysis showed that GNAS has a tendency to increase by threefold in F1 females (*p* = 0.0635) and a tendency to decrease in F3 females by 0.3-fold (*p* = 0.074) (Fig. 5b). In F1 males we observed a tendency to decrease in GNAS amounts by 0.2-fold in (*p* = 0.057) and a nonsignificant decrease in F3 was detected (Fig. 5b).

Next, we prepared tissue sections and performed immunofluorescent analysis of GNAS in females. The analysis showed that GNAS protein is mostly localized at the membranes. The analysis detected a significant increase of GNAS levels in F1, and a non-significant decrease in F3 (Fig. 5c,d), the directions of the changes are consistent with our western blot data.

### Gene expression analysis reveals dramatic changes in the expression of genes involved in thyroid development and hormone biosynthesis and transport

**Figure 6:**
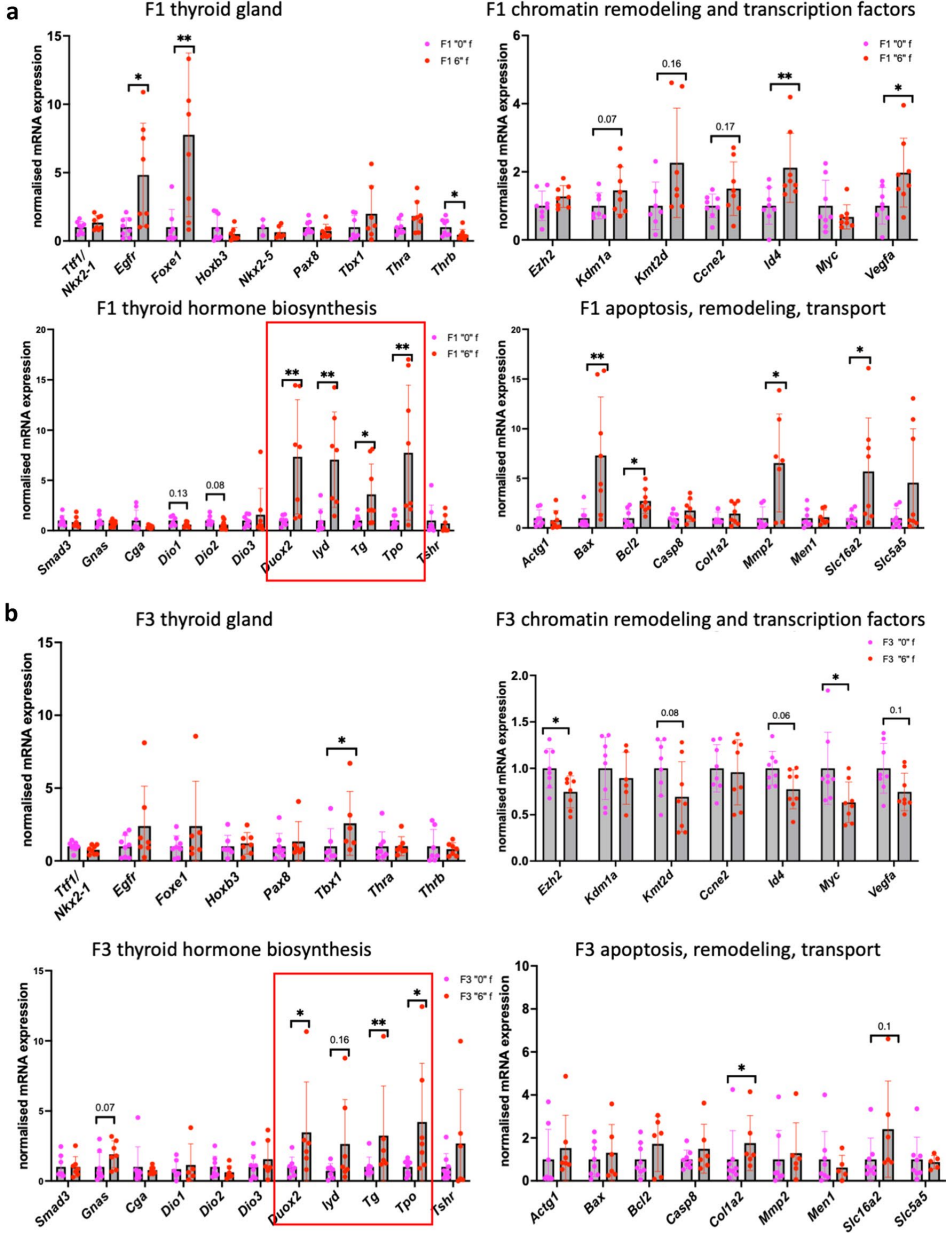
Gene expression analysis by RT-QPCR in the thyroids of F1 and F3 female mice. (**a**) Gene expression analysis in F1 and in (**b**) F3 female thyroid. The genes were grouped on the plot according their function. RNA was extracted, cDNA synthesized, diluted and used for qPCR. Each value was normalised to *Rpl13a* and *Rpl37a*. N = 8 for each group, *p < 0.05, **p < 0.01, Mann–Whitney test.

To reveal the effects of *thia* exposure on gene expression in the thyroid gland, we chose several target genes based on their importance for thyroid gland development, hormone biosynthesis, hormone transport and remodelling functions (Supplementary Fig. S3, Supplementary Table 1). We extracted RNA, synthesized cDNAs and performed RT-qPCR analysis in F1 and F3 groups from both sexes. In F1 females, we found that *Egfr* and *Foxe1,* which are important for thyroid gland development, were strongly upregulated by 4.8 and 7.8 folds, respectively. These changes were not significant in F3 (Fig. 6a,b). *Id4* was upregulated in F1 by 2-fold and decreased in F3 0.7-fold. Another factor, *Tbx1* significantly increased in F3 by 2.3-fold. The most dramatic changes in female thyroid were observed in thyroid hormone biosynthesis genes. We observed a dramatic increase in *Duox2, Iyd, Tg* and *Tpo* in F1 by 7.3, 3.6, 7.8-folds, respectively. *Duox2, Tg and Tpo* were significantly increased in F3 by 3.4, 3.2 and 4.2-folds respectively. *Slc16a2* encoding the transporter MCT8 has increased by 5.7-fold in F1 and tend to increase in F3 by 2.4-fold (Fig. 6a,b).

**Figure 7:**
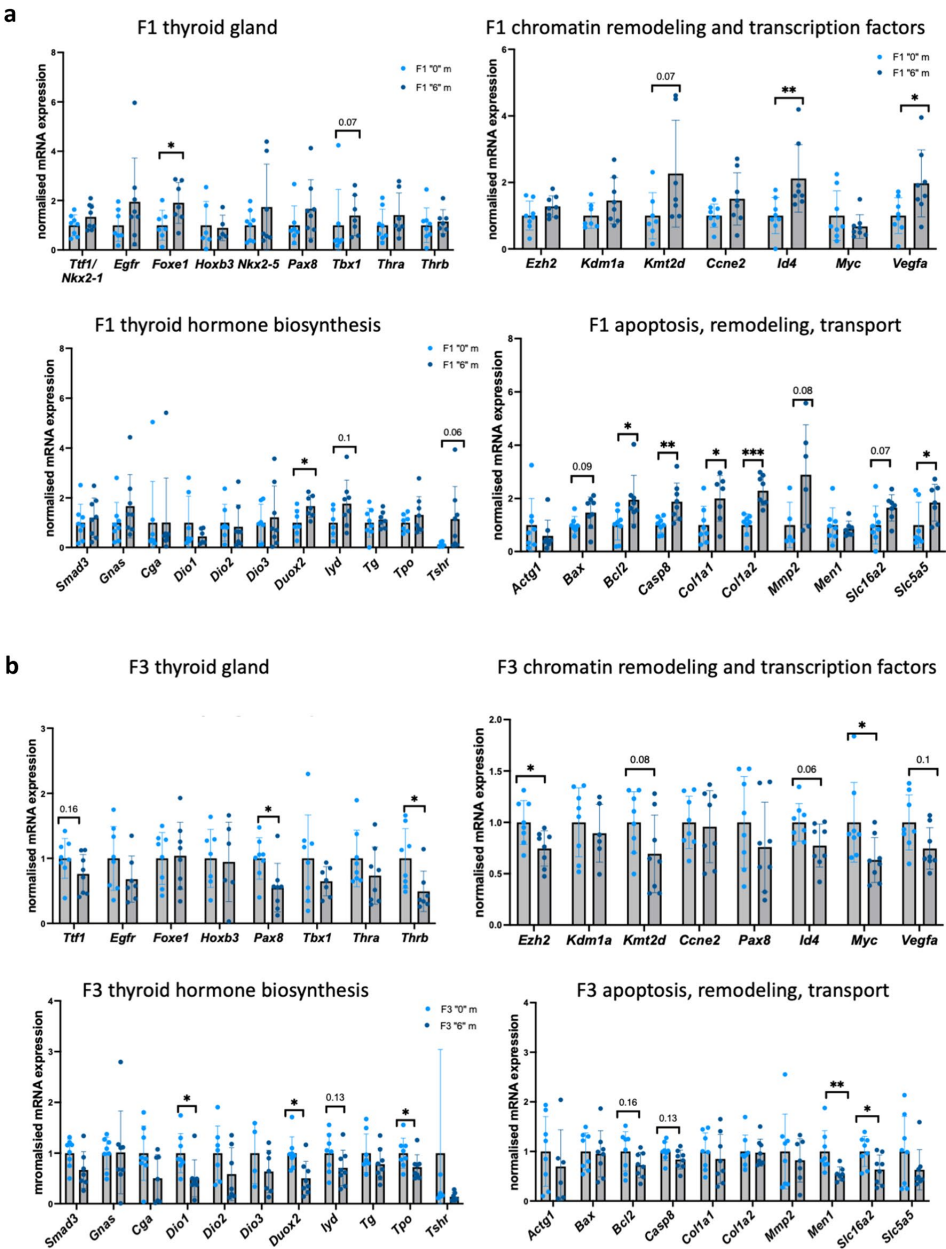
Gene expression analysis by RT-QPCR in the thyroids of F1 and F3 male mice. (**a**) Gene expression analysis in F1 and in (**b**) F3 male thyroid. The genes were grouped on the plot according their function. RNA was extracted, cDNA synthesized, diluted and used for qPCR. Each value was normalised to *Rpl13a* and *Rpl37a*. N = 8 for each group, *p < 0.05, **p < 0.01, Mann–Whitney test.

In F1 males, the expression of the *Foxe1* was similarly upregulated by 1.9-fold in the treated group while no changes were detected in F3. *Id4* was upregulated in F1 and decreased in F3. *Pax8* had decreased by 1.8-fold in F1. We observed a similar increase in *Duox2* by 1.6-fold in F1 and a decrease in *Duox2* and *Tpo* in F3 males by 2 and 1.6-fold, respectively. We also observed that gene expression of *Dio1* decreased in F3 males by twofold. *Slc16a2* had a tendency to increase in F1 by 1.6-fold and significantly decreased in F3 by 1.6-fold (Fig. 7a,b).

In conclusion, our analysis shows that there are significant changes in the expression of genes involved in thyroid development, hormone biosynthesis and transport in F1 and F3 thyroids from both sexes. The genes encoding proteins important for hormone biosynthesis were strongly upregulated in both F1 and F3 females which is consistent with the observed T4 hormone increase in females.

### Analysis of H3K4me3 occupancy in sperm revealed alterations at *Dio1*, *Mmp2* and *Tg* loci

**Figure 8:**
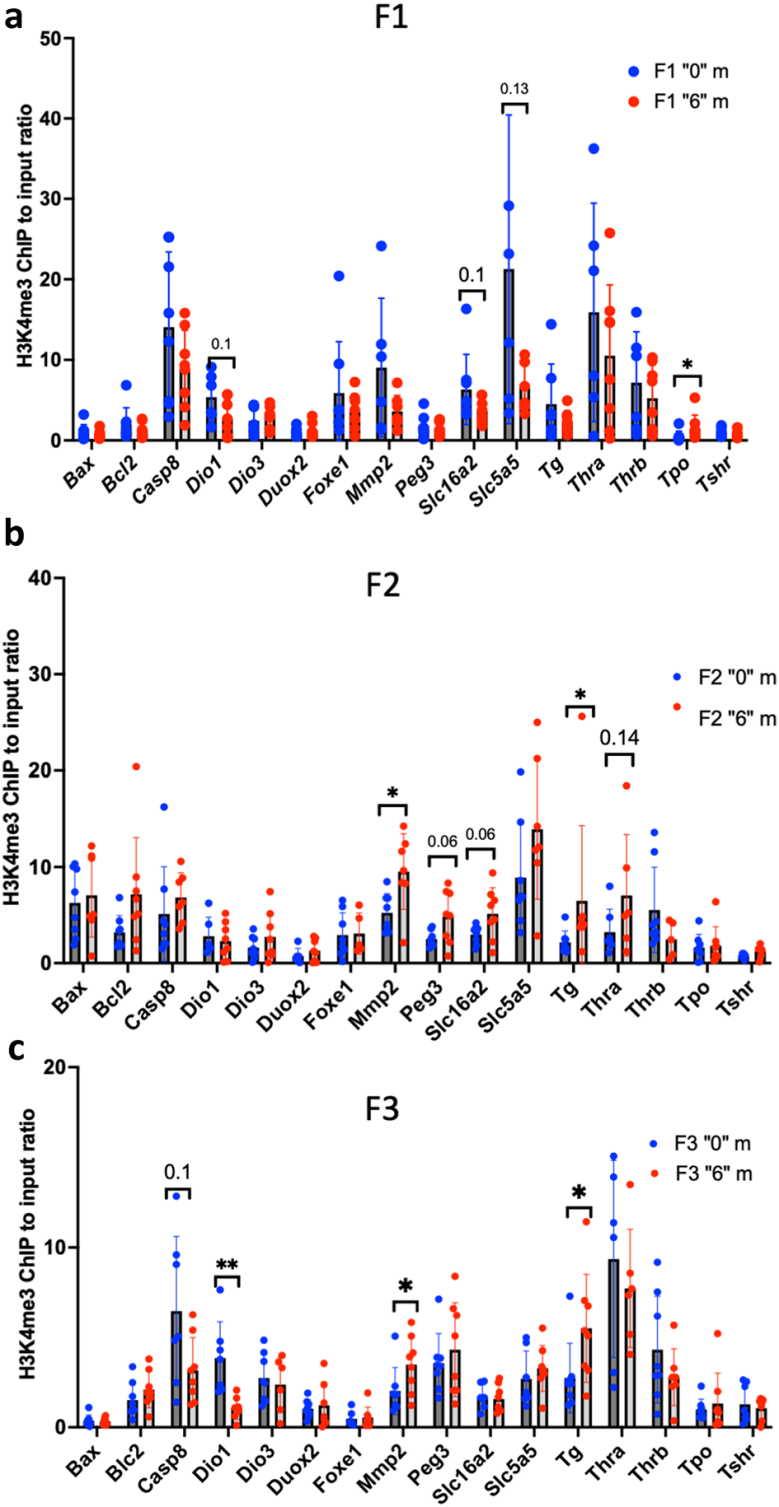
ChIP-QPCR analysis of H3K4me occupancy in the spermatozoa of F1 (**a**), F2 (**b**) and (**c**) F3 males. Sperm was extracted at the moment of dissection by piercing the cauda and incubated at 37C during 1 h allowing swim-out in DMEM media. Cells were washed, strained, pelleted and preserved at − 80°C until use. ChIP was performed as described in the "Methods" section, n = 6 for each group. *p < 0.05, **p < 0.01, Mann–Whitney test.

Because the previous results show that some genes have an altered expression in the third generation of exposed animals, we decided to look whether these expression changes have been transmitted through generations via epigenetic changes in sperm. It is suggested that regions which retained histones in the sperm are responsible of the transmission of the induced alterations to subsequent generations^32^. In order to check whether H3K4me3 marks are preserved in sperm, we used the most recent dataset of sperm H3K4me3 occupancy in mice^33^. We found that most of the thyroid genes we used in our gene expression analysis display H3K4me3 at their promoters except for *Tpo*. To reveal whether the histone containing fraction shows modified H3K4me3 occupancies, we performed ChIP followed by qPCR in F1, F2 and F3 sperm (Fig. 8a–c). An equal amount of immunoprecipitated DNA and Input were used for qPCR. We narrowed the analysis to the promoters of the genes that were differentially expressed in thyroid. In F1 sperm we did not observe a significant difference between the control and the treated groups except for a tendency to decrease for *Dio1* and *Slca16a2* promoters. In F2 sperm, the promoters of *Tg* and *Mmp2* genes have increased H3K4me3 occupancy and a tendency to increase for *Slc16a2,* and *Peg3*. *Dio1* has a tendency to have decreased H3K4me3 occupancy in F1 and have decreased occupancy in F3 which is consistent with *Dio1* protein levels in F1 and F3 in females and with gene expression in F3 males (Fig. 8a–c).

To conclude, our data shows that changes in gene expression of *Dio1, Mmp2* and *Tg* in F3 could be explained by increased histone H3K4me3 occupancy in the sperm at their promoters.

### DNA methylation analysis in sperm reveals changes in genes important for thyroid gland function

**Figure 9:**
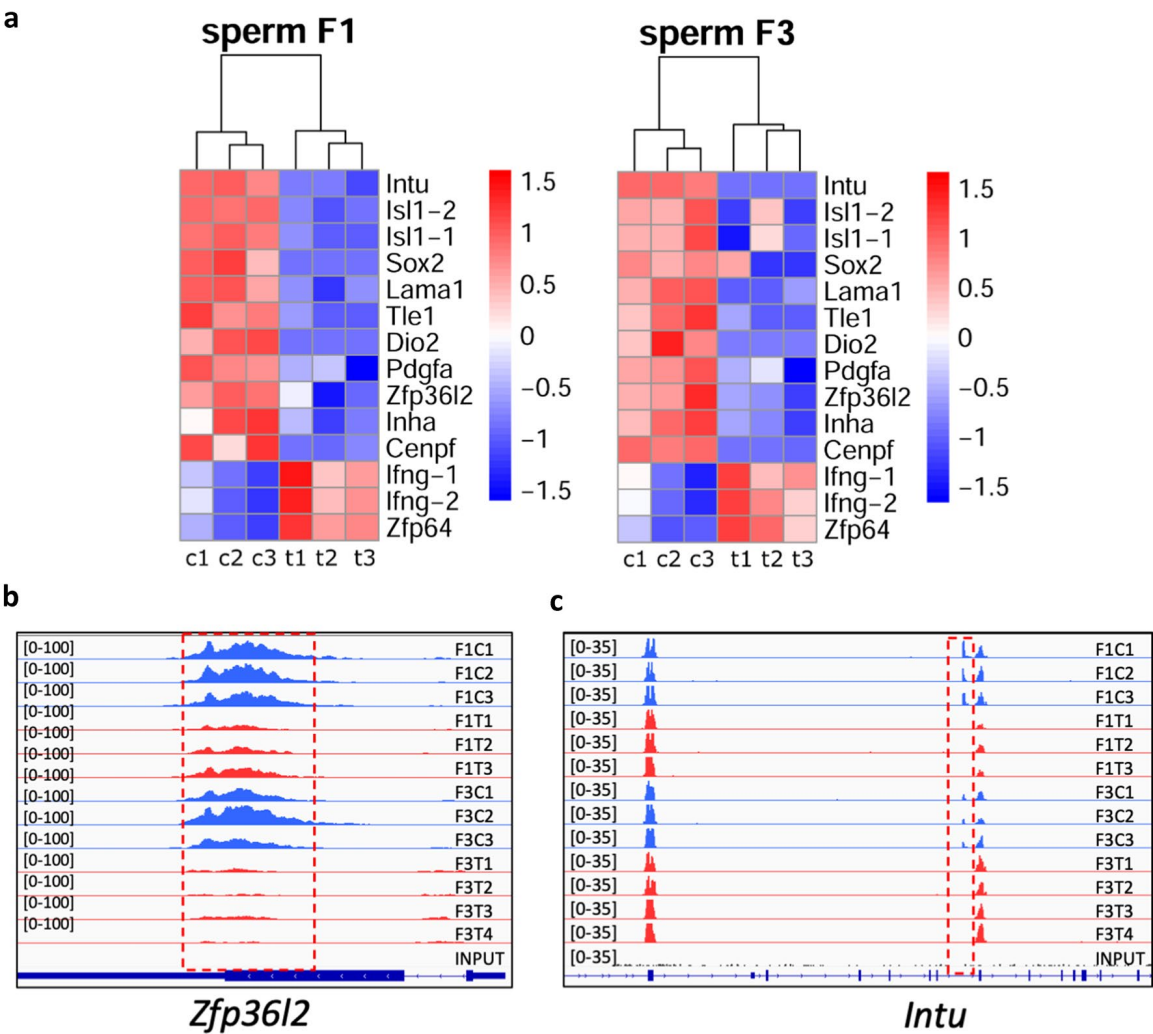
DNA methylation analysis in the sperm of F1 and F3 mice at genes that play a role in thyroid function. (**a**) Heatmap of DMRs. MeDIP counts with FC > 1.5 and FDR < 0.1 were log transformed and plotted in R using Pheatmap, c1–c3 are controls, t1–t3 are thia-derived samples. The plots of sequencing read of differentially methylated regions overlap (**b**) *Zfp36l2* and (**c**) *Intu* genes, the signal range is indicated in brackets. The sequencing analysis was performed using sperm DNA and a minimum of 3 replicates for each group in F1 and F3. Differential peaks are marked by red dashed boxes. The genes located in differentially methylated regions and related to thyroid were identified from the previously published litterature.

Besides histone modifications, sperm DNA methylation could also be involved in epigenetic control of gene expression and could play a role in the development of future organisms^34–36^. In our previous study, we analysed the effects of thiacloprid on sperm DNA methylation in F1 to F3 generations^26^. Although we did not detect a large number of DNA methylation changes that were consistent between F1 and F3, we still identified more than 30 regions that show a similar direction change between F1 and F3^26^. We looked closer at these regions and we found that changes in 15 regions could have an impact on thyroid function (Fig. 9a). For example, we found a decrease in DNA methylation at the *Zfp36l2* gene locus (Fig. 9b) and an effect on thyroid has been reported in the corresponding knock-out female mice^37^. We also found a decrease in DNA methylation in the gene body of the *Intu* gene (Fig. 9c) which was found to be downregulated in a human autoimmune thyroid disease^38^. The other genes whose DNA methylation was found dysregulated in our study include *Isl1, Sox2, Dio2, Lama1, Inha, Ifng, Cenpf, Tle1* (Fig. 9a).

## Discussion

### Effects on morphology

In this study, we aimed at revealing the effects of a widely used insecticide, thiacloprid, on the thyroid in directly exposed animals and in the descendants of exposed animals. The Environment Protection Agency (EPA) in the USA defines thiacloprid’s no-observed-adverse-effect level (NOAEL) dose in female mice is equal to 27.3 mg/kg/day and in males is 102.6 mg/kg/day. In France, daily authorised dose for human is 0.01 mg/kg/day. Dose “6” chosen in this study were 4.55 (female) and 17.1 (female) fold lower than the NOAEL dose and 600 higher than daily authorised dose for human in France. In our previous study^26^ we found that reproductive parameters such as spermatozoa numbers, density of cells, and testis weight were significantly altered only at the highest dose, 6 mg/kg/day, but not at “0.6” mg/kg/day. Thus, we suggested that the effects promoted by “0.6” mg/kg/day in F1 may have reverted in F3. The effects of low doses cannot be predicted by the effects observed at high doses^39^, thus low and high doses may induce different responses. The mechanisms of toxicity of thiacloprid are complex and could be induced by indirect alterations via effects on the central and peripheral nervous system. The screening of neonicotinoids binding to oestrogen and thyroid hormonal receptors showed a lack of agonist effects for any of the neonicotinoids tested^40^. Thus, it is likely that some neonicotinoids effects are indirect. Neonicotinoids would act on thyroid gland function via interfering with TSH production, which is, in turn, dependent of the HPA axis regulation.

TSH appears to play critical roles in thyroid biosynthesis. TSH-regulated activation of cAMP-dependent protein kinase has been demonstrated in calf thyroid slices^41^. TSH, via its receptor (TSHR), increases thyroid hormone levels by up-regulating expression of the sodium iodide symporter (*NIS/Slc5a5*), thyroid peroxidase (*TPO*), and *TG* genes. Considering importance of TSH in thyroid morphology one could imagine that the change in the genes important for the thyroid biosynthesis could explain the changes in T3/T4 hormone levels.

In this study, we did not measure T3 levels in serum. Changes in the T3/T4 secretion ratio could be affected by low thyroid *Dio1* protein activity. We report a decrease in H3K4me3 occupancy at the *Dio1* gene in the F3 generation and a tendency to decrease in *Dio1* (*p* = 0.12) gene expression in F1. Dio1 immunostaining shows a protein level decrease in *thia*-derived females (Fig. 4). This would indicate that the possibility of a posttranscriptional regulation of the *Dio1* gene products. The deficiency of DIO1 in the thyroid could be compensated by DIO2. We determined that the *Dio2* gene shows decreased DNA methylation in the sperm of F1 and F3 males. One can imagine that the observed decreased methylation at the *Dio2* locus in our study may be associated with an increased expression in other organs than thyroid. Indeed, *Dio2* is expressed in many tissues, including the central nervous system, pituitary gland, skin, retina, brown adipose tissue and skeletal muscle^42^. In, human only 20% of plasmatic T3 is derived from thyroid secretion, the remaining being produced outside the thyroid by DIO1 and DIO2^43^. We also observed a high expression level of the thyroxin transporter MCT8/*Slc16a2*. We can similarly propose that the transport of T4 may be increased which might explain the slight but significant increase of T4 that we detected in plasma. It is conceivable that, in order to compensate the low production of T3 in thyroid, DIO2 could be upregulated in other organs.

### Sex-specific changes

In F1 directly exposed females, we did not observe significant changes in the numbers of follicles or their volume but the overall weight of thyroid was decreased. We observed a high variation in the size of the colloids in the F1 treated group. We believe that the fact that colloids show such size heterogeneity upon the treatment also represents the aberrant phenotype in females. In F3 females, we observed a decrease in the size of colloids, similar changes were detected in F1 and F3 males. Thyroid development is regulated by transcription factors such as *Foxe1* and *Tbx1*, both of these factors show altered gene expression. *Foxe1* deletion in adult mice was associated with disruption in thyroid morphology and led to hypothyroidism, confirming its role in the maintenance of thyroid differentiation^44^.

We observed a stronger effect on genes involved in hormone biosynthesis (*Duox2, Iyd, Tg and Tpo*) in directly exposed females than in males suggesting that females might be more sensitive to exposure to thiacloprid. This is consistent with the fact that females tolerate lower doses compared to males based on EPA research^45^. On the other hand, females show a significant increase in T4 and TSH levels, suggesting that females may experience impact on thyroid mainly via the hormonal pathway. The difference could also be explained by an alteration in GNAS levels. By using a knockout mouse model, it has been shown that the metabolic phenotype generated by germline maternal loss of function of GNAS could be also reproduced by a loss of maternal GNAS expression in the central nervous system (CNS) only, suggesting that GNAS imprinting in the CNS underlies the parent-of-origin effect^46^. The study have shown that GNAS undergoes imprinting in the paraventricular nucleus of the hypothalamus, an area known to be important for metabolism central regulation^47^.

On the other hand, gonadotropin-releasing hormone (GnRH) neurons play a key role in the central nervous system regulation of reproduction. There is evidence for the involvement of the cholinergic system in the neuronal regulation of gonadotropin-releasing hormone neurons^48^. Since cholinergic receptors (*Chrna2, Chrna7, Chrnb2, Chrm4*) are upstream of G-proteins (*Gnas, Gnai1, Gnai2*)^49^ it is conceivable that alterations of cholinergic receptors may affect their downstream targets and, therefore, affect hormones production. These effects would be stronger in females due to leading maternal GNAS changes in the brain. The resistance to thyrotropin (TSH) action is a known human disease which is associated with molecular defects in the transmission of TSH signal into thyroid cells^50^. The defect may in principle affect every step along the cascade of events following the binding of TSH to its receptor (TSHR) on thyroid cell membranes. The mild hypothyroidism in autoimmune thyroid disease or pseudohypoparathyroidism is associated with genetic or epigenetic defects at the GNAS locus^50^. More recently, it has been reported that a mild TSH resistance may also be found in a subset of patients which shows a methylation defect at the GNAS locus^51^.

### Epigenetic effects

We observed that changes in epigenetic histone marks were inherited only in a few genes. Only 10% of the histones are preserved in the sperm^52^. Our analysis showed that out of 15 analysed targets regions, three of them have alterations in sperm H3K4me3 occupancy. These changes could be transmitted in the third generation via sperm inheritance and induce the changes in F3 thyroid.

On the other hand, we cannot exclude the fact that some changes could be derived from other somatic tissues, which are also impacted by alterations of paternal sperm. Both F3 male or females were generated from F2 males, which were derived from F1 males which were exposed in utero to thiacloprid which may have induced alterations in the germline. Indeed, 12 genes were found to be differentially methylated in the sperm of both F1 and F3 including *Isl1, Sox2, Dio2, Lama1, Inha, Ifng, Cenpf, Tle1* (Fig. 9a). These genes may play a role in the thyroid function. For example, mutations within Sox2/SOX2 are associated with abnormalities in the hypothalamo-pituitary–gonadal axis in mice and humans^53^. This axis is also essential for the production of TSH. *Zfp36l2* shows decreased DNA methylation in the sperm of F1 and F3 males. It has been shown that Zfp36l2 − / − females have decreased expression of the thyroid-specific transcripts and proteins, such as Nis/Slc5a5 and its transcriptional regulators, *Pax8 and Nkx2.1 37*.

## Conclusion

Our data suggests that ancestral exposure to neonicotinoids leads to transgenerational effects, possibly via inherited H3K4me3 and sperm DNA methylation, while thyroid function is perturbed via complex mechanisms that would include signalling through the hormonal axis.

### Limitations of our study

The use of outbred mice produces high heterogeneity in the results; thus, we had to increase the number of biological replicates for each experiment. The handling of such a high number of samples of both sexes led us to reduce our study to the effects induced by one dose only. We also chose to focus on analysing the genes important for the thyroid gland function via an RT-qPCR approach. This approach allows us to focus on a few genes and not to disperse on too many pathways, but it reduces our opportunity to detect new unknown genes involved in thyroid gland function. In this study, we did not study the maternal inheritance. Although, we acknowledge that thiacloprid could induce changes in the female germ line and the effects could be inherited by the future generations via the maternal germ line.

## Methods

### Ethics statement using animals

All experimental procedures using animals were authorized by Ministry of National Education and Research of France (Number APAFIS#17473-2018110914399411 v3). The animal facility used for the present study is licensed by the French Ministry of Agriculture (agreement D35-238-19). All experimental procedures followed the ethical principles outlined in the Ministry of Research Guide for Care and Use of the Laboratory Animals and were approved by the local Animal Experimentation Ethics Committee (C2EA-07). All methods were in accordance with ARRIVE guidelines. Most of animals were euthanised by a placing animal in carbon dioxide (CO2) chamber. For hormone measurement analysis mice were euthanised using 130mg/kg/body weight of ketamine (Virbac, France) and 13 mg/kg/body weight of xylazine/Rompun (Elanco, France). After blood was taken from the heart, mice were euthanised via decapitation. All procedures were authorised by Ministry of National Education and Research of France, the authorisation number is #17473-2018110914399411 v3. All euthanasia procedures were done according the annexe IV of low 2013-118 issued by Ministry of agriculture, food and forestry of France in a February 1, 2013.

### Mice treatment and dissection

Pregnant, outbred, Swiss (RjOrl, Janvier lab), female mice were treated with *thia* (0.06, 0.6, and 6 mg/kg/day) by administering the compound down the esophagus and into the stomach using a gavage needle from embryonic day E6.5 until E15.5, which corresponds to the somatic-to-germline transition window. The doses chosen in our study were determined according to the authorized daily dose (0.06 mg/kg/day) of another neonicotinoid, imidacloprid, established by the French Agency for Food, Environmental and Occupational Health and Safety (ANSES) and were 10 and 100 times higher than the authorized dose. After breeding, the day of vaginal plug detection was considered embryonic day 0.5 (E0.5). Thiacloprid (Fluka, R1628-100MG) was suspended in olive oil and administered in a volume of 150 µL for each mouse. The control mice were treated with the same volume of oil. These control and thia-treated mice are called F0. Progeny of exposed mice called F1. Both control and exposed F1 generation males were crossed with non-littermate and untreated female to give rise to F2 generation. Both control and exposed male progeny of F2 were crossed with non-littermate and untreated female to give rise to F3 generation. For each dose, a minimum of 10 unrelated pregnant female mice were treated, and for each assay, and 4–10 males from different litters were used for each assay. Animals from each generation were derived from two randomly chosen independent treatments. F1 and F3 generation male and female mice derived from treated and control groups at the age of 60 days were euthanized. The mice that were used in this study were 2-month-old. At this age, the thyroid organ is totally formed. Besides, male mice are sexually mature so spermatozoa were produced in sufficient amounts for the analysis.

### Morphology analysis

Thyroid glands from F1 and F3 mice were dissected and preserved in Bouin or paraformaldehyde at -80 °C. Then, the glands were fixed, included in paraffin and cut using a microtome to produce 5 µm sections. The sections were stained following the Hematoxylin and Eosin (H&E) staining protocol: first the slides were deparaffined using Shandon Varistain 24–3 stainer. Then, slides were immersed in distilled water for 1 min. Slides were then immersed in hematoxylin for 4 min then rinsed in running tap water for 1 min. Afterwards, slides were dipped in distilled water 4 times for 1 min each. Slides were then dipped in 100% alcohol containing 1% acetic acid for 15 s and then 5 times in distilled water. Next, slides were dipped in 3% ammonia (NH_4_OH) for 1 min and then dipped 5 times in distilled water. Then, slides were immersed in eosin for 2 min. Next, slides were dipped 2 times in 70% ethanol, then 5 times in 95% ethanol, then 1 min 30 s in 100% ethanol and, finally, 1 min in xylene 1 and 2 min in xylene 2. Slides were left to dry for a few minutes before mounting with Eukitt mounting medium. Slides were then scanned using Hamamatsu NanoZoomer slide scanner. The images obtained were used for morphology analysis using ImageJ. Image analysis consisted in measuring the area of a section, the number of follicles per section and the area of each follicle. Then, we calculated the radius, the average radius and the volume of each colloid following the method described in^54^ in which we considered the colloids as spheres. The formulas used were the following: $R = \sqrt {\frac{Area}{\pi }}$ where R is the Radius of the follicle and $R\left( m \right) = R\frac{4}{\pi }$ where R(m) is the average radius. This is to compensate for the effects of follicular cross-section. We also used $V = \frac{4}{3}\pi R^{3}$ where V is the volume of a follicle. For immunofluorescence experiments, the thyroids from 6 control and 6 *thia*-treated groups were fixed in 4% (w/v) PFA solution for 16 h, dehydrated and embedded in paraffin. The sections were cut with a microtome at a 5 µm thickness. The sections were deparaffinized, rehydrated, and immunostained according to a standard protocol. Statistical significance was assessed with a nonparametric Mann–Whitney test.

### Immunofluorescence on paraffin sections

For immunofluorescence, the epitopes were unmasked in 0.01 M citrate buffer, pH 6 at 80 °C for 20 min. After blocking in 4% BSA containing 1X PBS-0.05% Tween (PBS-T), the sections were stained with rabbit anti-DIO1 antibodies 1:200 (11,790–1-AP, Proteintech) or rabbit anti-GNAS antibody 1:200 (10,150–2-AP, Proteintech). The sections were incubated with primary antibody overnight at 4 °C in a humidified chamber. After washing in PBS-Kodak Photo Flo 0.04% solution, the sections were incubated with the appropriate fluorescent Alexa conjugated secondary antibody (1:1000, Invitrogen) for 1 h in a humidified chamber at room temperature. The sections were mounted using Vectashield mounting medium containing 0.001% (v/v) 4,6-diamidino-2-phenylindole dihydrochloride (DAPI). The images were taken using an AxioImager microscope equipped with an AxioCam MRc5 camera and AxioVision software version 4.8.2 (Zeiss, Le Pecq, France) with a 20X or 40X objective lens. Negative control stained with the secondary antibody only is displayed on Fig. S4.

### RNA extraction and RT-qPCR

Thyroids and brains were stored at − 80 °C. 60-day-old mice from the control “0” group and from the “6” mg/kg/day treatment group were used for RNA extraction. Extraction was performed using RNeasy Plus Mini Kit (2,552,951, QIAGEN^®^). Approximately 10 mg of each thyroid or brain structures were used. Thyroids or brain samples were lysed and subsequently homogenized using a TissueLyser (Qiagen) and 5 mm stainless-steel beads (69,989, Qiagen). DNA was removed by passing the solution through a DNA elimination column. One volume of 70% ethanol was added to the lysate to provide ideal binding conditions. The lysate was then loaded onto the RNeasy silica membrane. RNA was additionally treated with DNAse using RNAse free DNAse set (79,254, Qiagen) directly on the column. RNA was then washed with RW1 and RPE from Qiagen kit to remove impurities. Purified RNA was then eluted in 50 µL of RNase-free water. Eight biological replicates from controls and eight from treated groups were used for RT-qPCR for each group. RT was performed using 1 µg of total RNA with iScript (1,708,891, BioRad) adhering to the Minimum Information for Publication of Quantitative Real-Time PCR Experiments (MIQE) guidelines^55^. We used *Rpl37a* and *Rpl13a* as housekeeping gene as, based on our RNA-seq data it showed no variation between replicates. The data were presented as fold changes compared to control + /−SD. Primers for this study were selected using Primer-Blast program from *ncbi.nih.gov* and most of them include exon-to-exon junctions. Primers used in this study are listed in Supplementary Table 2. A nonparametric Mann–Whitney test was used for statistical significance.

### Chromatin immunoprecipitation (ChIP)

We performed ChIP using rabbit polyclonal antibodies against H3K4me3 (07-473, Milipore). Equal amounts of material (~ spermatozoa from one mouse) were used and incubated in 1% paraformaldehyde solution for 10 min to crosslink proteins to DNA. 100 μL of 1.25 M Glycine were added to each sample to quench the unbound paraformaldehyde. The samples were centrifuged, and 1 mL of PBS and two metal beads were added to the pellet which was homogenized using TissueLyser (Qiagen). Then, the samples were filtered in a cell strainer and the resulting solution was pelleted and resuspended in the following buffer: 0.25% (vol/vol) Triton X-100, 10 mM EDTA, 0.5 mM EGTA, 10 mM Tris pH8. Samples were centrifugated at 1100 g for 5 min, 4 °C and the pellets, containing cells, were resuspended in 300 μL of SDS lysis buffer (1% (wt/vol) SDS, 10 mM EDTA, and 50 mM TrisCl pH8) in the presence of a protease inhibitor and 10 mM DTT and incubated during one hour at RT. Chromatin was sonicated in SDS lysis buffer at 60% amplitude for 8 min (20 s on, 20 s off), using Qsonica 700 sonicator (Q700-110, Newtown, Connecticut, USA) supplied with cup horn 431C2; these parameters allow to obtain ~ 300 bp chromatin fragments. After sonication, samples were centrifugated at 12,800 rpm for 10 min at 4 °C and the supernatant containing sonicated chromatin was transferred and diluted in 1.7 mL of the following buffer: 0.01% (1.1% (vol/vol) Triton X-100, 1.2 mM EDTA, 16.7 mM TrisHCl, 167 mM NaCl. A solution containing 20 μL of Dynabeads (10002D, Invitrogen) and an 0.7 µL of anti-H3K4me3 antibody (07-473, Millipore) was added to the sample tubes and incubated overnight at 4 °C. Before adding the antibody and Dynabeads, 10 μL of each sample were collected as “Input samples” (starting material). After overnight incubation with Dynabeads and the antibody of interest, the beads were washed 5 min each, with the following four buffers: (1) Low salt buffer: 0.1% (wt/vol) SDS, 1% (vol/vol) Triton X-100, 2 mM EDTA, 20 mM TrisHCl, 150 mM NaCl; (2) High salt buffer: 0.1% (wt/vol) SDS, 1% (vol/vol) Triton X-100, 2 mM EDTA, 20 mM TrisCl pH8, 500 mM NaCl; (3) LiCl buffer: 0.25 M LiCl, 1% (vol/vol) Igepal, 1 mM EDTA, 10 mM TrisCl, pH8, 1% (wt/vol) deoxycholic acid; (4) TE buffer (two washes). Following the washing steps, the beads were resuspended two times in 50 µL of 1% (wt/vol) SDS, 0.1 M NaHCO3 pH9 and incubated at 65 °C for 15 min to elute the precipitated chromatin from the beads. Subsequently, the eluted chromatin was reverse cross- liked by adding 9 µL of 5N NaCl and incubating at 65 °C for 4 h. Then, proteins were removed by adding 1 µL 20 mg/ml of proteinase K and incubating the samples for 1 h at 45 °C. The precipitated DNA was purified using MiniElute Reaction Clean-Up kit (Qiagen) and the DNA concentration was measured using QuantiFluor dsDNA system (Promega). A minimum of ~ 3 ng of DNA was obtained.

### ChIP-qPCR

Equal amounts of precipitated DNA and input samples were used for the qPCR analysis. Quantitative PCR was performed using 0.4 ng of DNA of immunoprecipitated or Input DNA and 6 biological replicates. Normalized expression values were calculated with CFX Manager program using a region located far from promoter as a reference gene, we used a region in *Rplpo* for H3K4me3-ChIP normalization. Primers used in this study are listed in Supplementary Table 3. Enrichment of each target in the precipitated DNA was evaluated by calculating the ratio between the average of the normalized ChIP DNA copies and the average of the normalized DNA copies in the inputs.

### Thyroid hormone T4 and TSH measurement by ELISA

The blood was allowed to clot by leaving it undisturbed at room temperature. This usually takes 15–30 min. We removed the clot by centrifuging for 10 min in a refrigerated centrifuge. The resulting supernatant was aliquoted and preserved at − 80 °C until use. We used mouse Thyroid Stimulating Hormone ELISA Kit (MBS2700156-96, CliniSciences) and Mouse Thyroxine ELISA Kit (NBP2-60,162, Biotechne) for hormone measurement. We followed the instruction provided by the supplier. The data were averaged and plotted and presented as ng/ml.

### Western blot

Western blots were performed using a rabbit 1:1000 anti-GNAS polyclonal antibody (10,150–2-AP Proteintech). Equal amounts of protein extracts (10 μg) in 10 mM Tris buffer and Laemmli 4X buffer were denatured and ran on a 4–15% gradient SDS-PAGE gel (Mini-PROTEAN^®^ TGXTM Precast Protein Gels). Proteins were transferred onto Polyvinylidene difluoride (PVDF) membranes (Millipore, France) using an electro-blotter system (TE77X; Hoefer, USA) for 1.15 h. Blocking was conducted using a 5% milk in 1X TBS Tween 0,05%. The primary antibody was diluted in 10 mL of the blocking solution and the membranes were incubated overnight at 4 °C. After three ten-minutes washes with 1X TBS, each membrane was incubated for 1 h in 40 mL of the blocking solution containing the corresponding HRP-conjugated secondary antibodies (1:10,000, GE Healthcare, USA). After another three ten-minutes washes with 1X TBS, western blotting detection reagents Amersham ECL™ Prime Western Blotting Detection Reagent (RPN2232, Amersham) were used to coat each membrane. Specific protein expression for the antibody was then detected and measured using a molecular imager (ChemiDocTM XRS + System with Image LabTM Software). Ponceau Red- stained bands were used to assess protein loading and normalize the levels of GNAS for each sample. Intensity of the bands were measured using Fiji: ImageJ software.

### Statistical analyses

We used the minimum number of animals according to the requirements of the EU ethic committee. The number of animals used was specified for each experimental procedure. We performed non-parametric Mann–Whitney tests to assess statistical significance in qPCR experiments, immunofluorescence and western blots quantifications. Non-parametric Kruskal–Wallis test was used to access the statistical significance in body weight measurements.

### Supplementary Information

Supplementary Information.

## Data Availability

All data generated or analysed during this study are included in this published article and its supplementary information files.
