# Supplementary material for: Sex-specific transgenerational effects on murine thyroid gland imposed by ancestral exposure to neonicotinoid thiacloprid
Source: Sci Rep. 2024 Jun 6;14:13047. doi: 10.1038/s41598-024-63986-w (PMC11156953; doi:10.1038/s41598-024-63986-w)
Supplement: Supplementary file 1 — Supplementary Information. [file 41598_2024_63986_MOESM1_ESM.pdf]

Sex-specific transgenerational effects on murine thyroid gland imposed by ancestral exposure to neonicotinoid thiacloprid

Mariam Diba Lahmidi[1]*, Morgane Le Noc[1]*, Ouzna Dali[1]*, Pierre-Yves Kernanec[1], Pierre-Etienne Merret[1], Christian Jaulin[1] and Fatima Smagulova[1]#

* These authors equally contributed to this work

1 Univ. Rennes, EHESP, Inserm, Irset (Institut de recherche en santé, environnement et travail) - UMR_S 1085, F-35000, Rennes, France

# Corresponding author

Supplementary Information

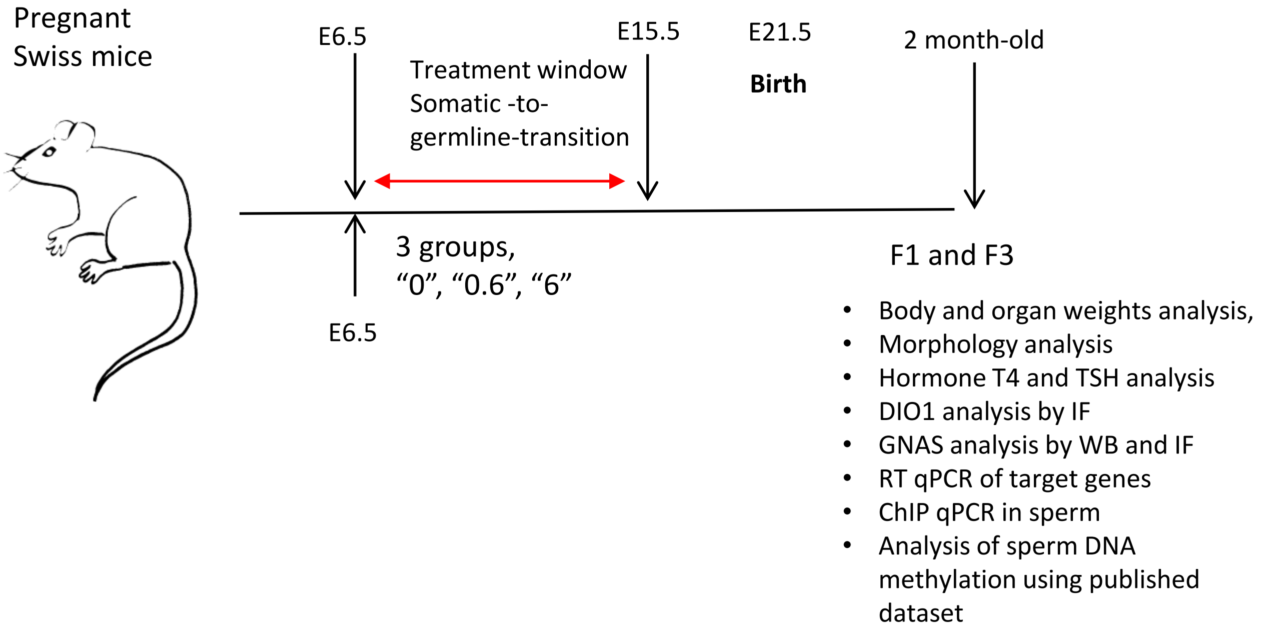

Supplemental Figure S1. Schematic presentation of experiments. Pregnant outbred Swiss mice were treated from E6.5 to E15.5, with *thia* doses of 0, 0.6, and 6 mg/kg/day, and control mice received only vehicle (oil). F1 and F3 mice were sacrificed at the age of 2 months. For spermatozoa ChIP-qPCR and F2 mice were also sacrificed. Figure was generated by PowerPoint. Image of the mouse is personal drawing.

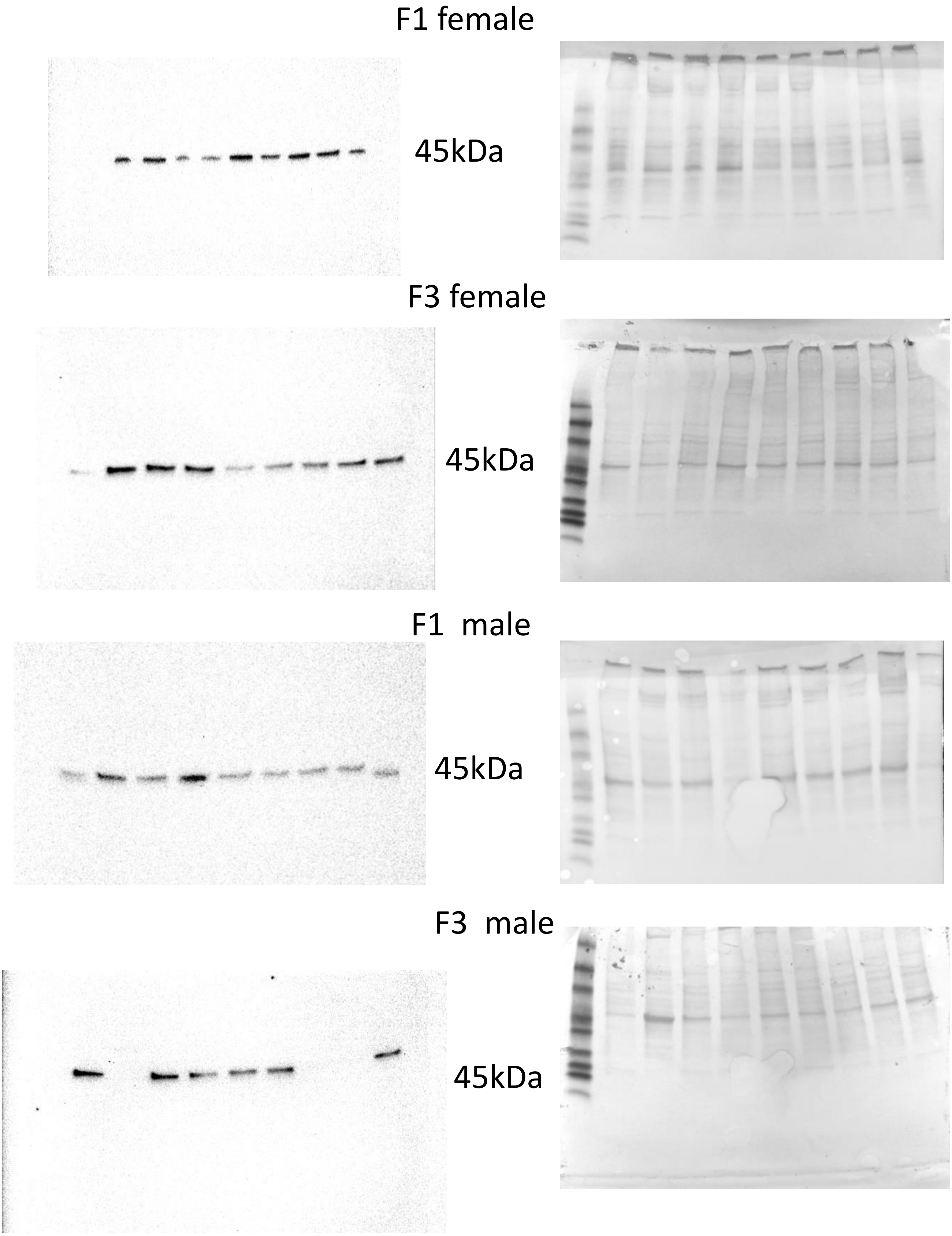

Supplemental Figure S2. Row uncut western blot pictures

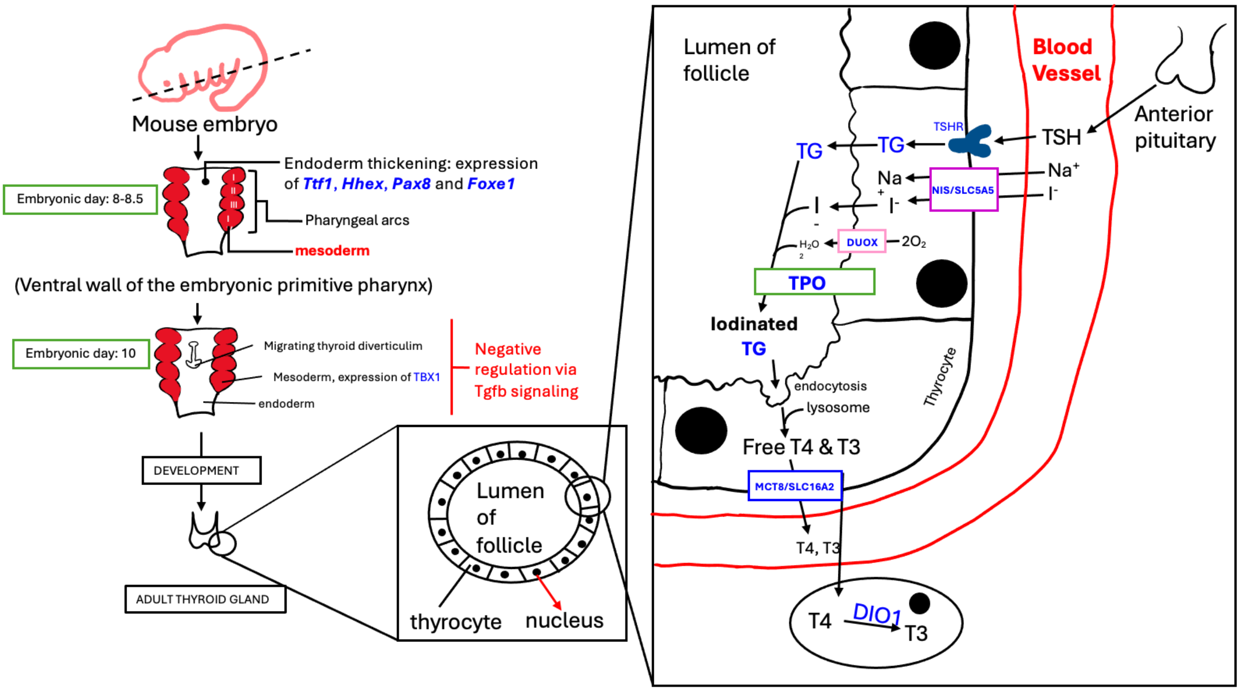

Figure S3. Development and functioning of the murine thyroid gland. Thyroid gland develops starting from embryonic day 8 with the expression of *Ttf1, Hhex, Pax8* and *Foxe1* genes. Thyroid proliferation is negatively regulated by Tgfb1 signaling. In adults, the thyroid follicular cell is called thyrocyte. In the thyrocyte, the formation of thyroxine T3 is induced under the regulation of the hormone TSH produced by the pituitary gland. TSH acts via binding to its receptor TSHR which is integrated into the membrane. TSHR stimulates the production of TG. Transporter NIS/SLC5A5 transfers I- into the cell. DUOX2 produces H_2O_2 which is needed to produce iodinated TG. TPO produces iodinated TG using I- and H_2O_2 in the lumen. Iodinated TG is integrated into the cell via endocytosis where is degraded into T3 and T4. T3 and T4 are transported to blood vessels via transporter MCT8/SLC16A2. T4 is converted to T3 by deiodinases which are expressed in thyroid and other organs. Power point from Microsoft office was used to generate figure as well as (1) and (2).

1. Mario De Felice, Roberto Di Lauro, Thyroid Development and Its Disorders: Genetics and Molecular Mechanisms, *Endocrine Reviews*, Volume 25, Issue 5, 1 October 2004, Pages 722–746

2. Löf C, Patyra K, Kero A, Kero J. Genetically modified mouse models to investigate thyroid development, function and growth. Best Pract Res Clin Endocrinol Metab. 2018;32:241–56.

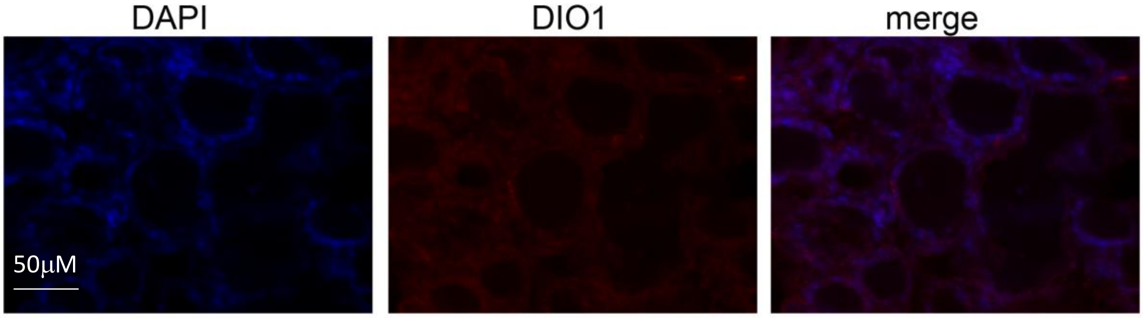

Figure S4. Second antibody only staining control for DIO1.

Supplementary Table 1. Target genes analysed in our study

ID	Gene Name	GOTERM_BP_DIRECT
Bax	BCL2-associated X protein(Bax)	apoptotsis process signaling
Casp8	caspase 8(Casp8)	apoptotsis process signaling
Bcl2	B cell leukemia/lymphoma 2(Bcl2)	apoptotsis process signaling
Thra	thyroid hormone receptor alpha(Thra)	regulation of thyroid hormone mediated signaling
Thrb	thyroid hormone receptor beta(Thrb)	regulation of thyroid hormone mediated signaling
Actg1	actin, gamma, cytoplasmic 1(Actg1)	regulation of thyroid hormone mediated signaling
Nkx2-5	NK2 homeobox 5(Nkx2-5)	thyroid gland development
Tbx1	T-box 1(Tbx1)	thyroid gland development
Cav1	caveolin 1, caveolae protein(Cav1)	thyroid gland development
Egfr	epidermal growth factor receptor(Egfr)	thyroid gland development
Fgf8	fibroblast growth factor 8(Fgf8)	thyroid gland development
Foxe1	forkhead box E1(Foxe1)	thyroid gland development
Hoxb3	homeobox B3(Hoxb3)	thyroid gland development
Pax2	paired box 2(Pax2)	thyroid gland development
Pax8	paired box 8(Pax8)	thyroid gland development
Smad3	SMAD family member 3(Smad3)	thyroid hormone biosynthesis
Gnas	GNAS (guanine nucleotide binding protein, alpha stimulating)	thyroid hormone biosynthesis
Dio3	deiodinase, iodothyronine type III(Dio3)	thyroid hormone biosynthesis
Dio2	deiodinase, iodothyronine, type II(Dio2)	thyroid hormone biosynthesis
Iyd	iodotyrosine deiodinase(Iyd)	thyroid hormone biosynthesis
Tg	thyroglobulin(Tg)	thyroid hormone biosynthesis
Tpo	thyroid peroxidase(Tpo)	thyroid hormone biosynthesis
Duox2	dual oxidase 2(Duox2)	thyroid hormone biosynthesis
Cga	glycoprotein hormones, alpha subunit(Cga)	thyroid hormone biosynthesis regulation
Tshr	thyroid stimulating hormone receptor(Tshr)	thyroid hormone biosynthesis regulation
Slc16a2	solute carrier family 16 (monocarboxylic acid transporters), member 2	thyroid hormone transport
Slc5a5	solute carrier family 5 (sodium iodide symporter), member 5	thyroid hormone transport
Men1	multiple endocrine neoplasia 1(Men1)	tissue remodeling
Col1a2	collagen, type I, alpha 2(Col1a2)	tissue remodeling
Mmp2	matrix metallopeptidase 2(Mmp2)	tissue remodeling

Supplementary Table 2. Primers used for RT-qPCR analysis

gene	forward	reverse
Bax	TTTGCTACAGGGTTTCATCCAG	CCAGTTCATCTCCAATTCGCC
Actg1	CTGGCACCACACCTTCTACA	GGAACAGAACCCTGCGTCAT
Bcl2	GTGTGGAGAGCGTCAACAGG	CACAAAGGCATCCCAGCCTC
Casp8	GGAACAGACTGTGATAAAGAGGCT	TTGTGGTCTGCGCTTTGGTA
Cga	CCTCAAAAAGTCCAGAGCTTGCAG	AGTTTACATTCTGGGCAACCCTG
Chrna3	TGGAAACCAACCTGTGGCTGA	TCCCCATCGGCGTTGTTGTA
Chrna7	GCCAGTATCTCCCTCCAGGCAT	AGGTCCAAGGACCACCCTCC
Chrnb2	TCCTTTGGCTGTGTTCAGGGGT	AGCTGGACGGATCAGCTTGTT
Col1a1	CAGGGATCCAACGAGATCGAG	GGGCCATATGTCTAGTCCGAA
Col1a2	AGGCTGACACGAACTGAGGTA	ATGCACATCAATGTGGAGGA
Dio1	GACAGGGCTGAGTTTGGGGG	GGGGCCTGCTGCCTTGAAT
Dio2	CCGCCCCAGTGTCAAGTTGT	GGGGCCCTTCCCTCCTAAGT
Dio3	CCAGCGCATCCTCGACTACG	GTCAACGTCGCGCTGGTACT
Duox2	ACCGGACTCCTGGGAACAGT	AGGGCCCCATTACCTTTTTGCC
Egfr	GTCTGCCACCTATGCCACGC	TCCACTGCCATTGAACGTACCC
Fgf8	CCAAGCCCAGGAAGGCCC	GGCTCTGCTCCCTCACATGC
Foxe1	CCGGGTAGGCACGAAACCTC	ACAGTGCAAGACACCGAGCC
Fshb	CAGCTGACTGCACAGGACGTA	CAGCTATGGCAGCAGATTGCTCT
Gnas	TCTCTTCCTAACAACAAGAC	GATTCAGAAACTCATCCCGA
Hoxb3	GACCGGCTGTTGGGGGAAAA	CTGAGCTCCTCTGGTCCCGT
Iyd	CACCGCCCCAGTTCTGATCC	CGTCACTAGCCCTGCATTCTGC
Men1	TTCCCAGCAGGCAGTGCG	CGGGCGGTGGCATCC
Mmp2	AGAAGAAAATGGACCCCGGTT	GCCCAGCCAGTCTGATTTGA
Nkx2-1	TCGGAAGGGAAAACTGCGGG	ATGATTCGGCGTCGGCTGGT
Nkx2-5	CTCTCCTGCTTTCCCAGCCG	CAGCGCGCACAGCTCTTTTT
Pax2	CCCTAGCTACCCGAGGGTCC	GCCGTGTCGCCTCTCAAACT
Pax8	CAGGCATGGTGGCAGGAAGTG	TGGTGCGCTTGGCCTTGATG
Slc16a2	TGCAACGGCTCCATCTTCGG	GCTCCGACCCATGCTGCTT
Slc5a5	GGGACGCTGCAGTACTTGGT	ATGTCCAACCCGGTCACTTGG
Smad3	GTTGGAAGAAGGGCGAGC	AGGCGGCAGTAGATAACGTG
Tbx1	CACCAAGGCAGGCAGACGAA	AGCGCTTGTCATCTACGGGC
Tg	TGGCCATGGCAGCCTAGAAT	AGGCTCTGCTCCTCCGTTGA
Thra	CCCAAGCTGCTGATGAAGGAGAG	ACCTGCGGACCCTGAACAAC
Thrb	GCTCCAGCGCTCTGATCCGT	CTGGCATTCCCTCTGACCTTCT
Tpo	GACCACCCAGTCAGGATGCAG	ATCCACTTGCAGGTTCCTGTCC
Tshb	AACGGAGAGTGGGTCATCACAG	GGCAGCACTCATGCTCTCCAC
Tshr	ACAGTGGACCAACTTTGCTGTCA	GTGGGGCATAAGGACGGCAG
Rpl13a	AACGGACTCCTGGTGTGAAC	TGGTCCCCGCTTCCCTAGTT
Rpl37a	TGGGGCCTGGACCTACAA	GCAGGGCTTCTACTGGTCTT

Supplementary Table 3. Primers used for ChiP-qPCR analysis

gene	forward	reverse
Bax	TCCTAGGGTTCTTGGACCGGG	TCACGTGACCGTGGTGCG
Bcl2	GGGCAGAGAGACACGCCAAG	CGTTTCGGAAAGCGCGTTGG
Casp8	AGGTACTCGGCCACAGGTGA	ACCCCGATGGCTGCATTTCG
Dio1	GAATTCGCCCCACTTCGCCT	TCACCCAGCGAAGACTCGGA
Dio3	TGGGACCTAGGAGGTGCGAG	GAAGCCGCTGTGTCCAGGG
Duox2	GATCACGCCTCTGCGCTTCT	CCCACGCAGTCATCCTGCAA
Foxe1	GCCGCCCTACAGCTACATCG	ACTTCTTGGGGTTGTCGCGG
Mmp2	GTAAGGGGATCGCCGTGCAG	ACTCGTGCCTCCATCGTTGC
Peg3	CTGCGGCAAAACAAGACCCG	CGGTTAACCCGATCGCCAGG
Slc16a2	GATCGAACGGCCTGACTGG	TCGCTCAGAGTCAACACATGG
Slc5a5	CCGGTGAAGAAGTCGTCGG	GGGACTACGGCGTGTTCG
Tg	CCTCCGGAGTGCGTTTTCAC	CTTTGACAGGCTCGGGGTG
Thra	CGTCGTTGCTCAGCTCGGA	AGACCCGCGGCTTTGCATAAT
Thrb	GTGACGCGGCGGGATTAACT	GCCCACCCTGCCAAGTTACC
Tpo	GTCTCCAGCCAGGAAGCAG	CTGCGTGTCAACACTCTCCA
Tshr	GGGTGTCTCGGAACAGAAGG	CTGGAGAGGGGGAAGGAGAG
Rplp0	ACTGGTCTAGGACCCGAGAAG	TCAATGGTGCCTCTGGAGATT
